# Crohn’s and Parkinson’s Disease-Associated LRRK2 Mutations Alter Type II Interferon Responses in Human CD14^+^ Blood Monocytes Ex Vivo

**DOI:** 10.1007/s11481-020-09909-8

**Published:** 2020-03-16

**Authors:** Tsuneya Ikezu, Lacin Koro, Benjamin Wolozin, Francis A. Farraye, Audrey J. Strongosky, Zbigniew K. Wszolek

**Affiliations:** 1 Department of Pharmacology and Experimental Therapeutics, Boston University School of Medicine, Boston, MA 02118 USA; 2 Department of Neurology, Boston University School of Medicine, Boston, MA 02118 USA; 3 Section of Gatroenterology, Inflammatory Bowel Disease Center, Boston University School of Medicine, Boston, MA 02118 USA; 4 Present Address: Departments of Gastroenterology and Hepatology, Inflammatory Bowel Disease Clinic, Mayo Clinic Florida, Jacksonville, FL 32224 USA; 5 Department of Neurology, Mayo Clinic Florida, Jacksonville, FL 32224 USA

**Keywords:** Crohn’s disease, LRRK2, Monocytes, Parkinson’s disease, Single nucleotide polymorphism, Type II interferon

## Abstract

The Leucine Rich Repeat Kinase 2 (*LRRK2*) is one of causative genes of familial Parkinson’s disease (PD). The M2397T polymorphism in *LRRK2* is genetically associated with sporadic Crohn’s disease (CD). LRRK2 is expressed in human CD14^+^ monocytes, induced by interferon-γ (IFN-γ) and suppresses inflammatory activation. We hypothesize that IFN-γ-induced LRRK2 and inflammatory gene expression is altered by *LRRK2* genetic polymorphism found in CD and PD cases. A total of 46 CD and 51 control cases, and 16 PD cases and 16 PD-linked *LRRK2* mutation cases were recruited. Live human CD14^+^ monocytes were isolated from donors for ex vivo IFN-γ stimulation and gene expression analysis. IFN-γ potently enhanced *TNFA, IL12, HLADRA1 and LRRK2* expression, which was suppressed by FK506, a calcineurin-specific inhibitor, but further enhanced by LRRK2-specific kinase inhibitor (GSK2578215A). The 2397-M/M CD risk allele enhanced IFN-γ responses of CD14^+^ cells in CD but not in control group. CD14^+^ monocytes from G2019S and R1441C *LRRK2* mutated PD cases and carriers show no changes in IFN-γ responses for *TNFA* or *IL12*, reduced response for *HLADRA1,* and enhanced responses for *LRRK2* in FK506-sensitive manner. These data demonstrate that CD-associated *LRRK2* mutations are significant modifiers of innate immune response in CD14^+^ monocytes, and PD-associated *LRRK2* mutation may contribute to reduced antigen presentation response.

**Figure:**
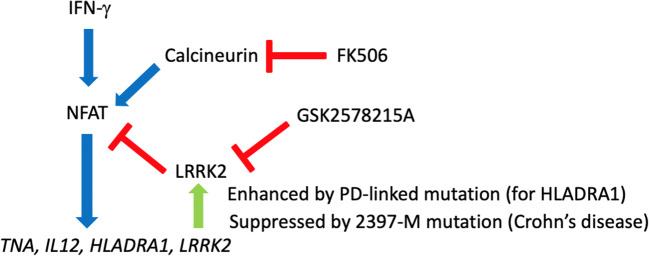
Graphical Abstract

Graphical Abstract

## Introduction

The Leucine Rich Repeat Kinase 2 (*LRRK2*) gene is a causative gene of familial PD (Cookson 2010). It has a kinase domain, which shares homology with the RIP and MKKK family of kinases (Cookson 2010). *LRRK2* has been identified as the gene mutated most frequently in autosomal dominant familial Parkinson’s disease (PD). PD patients with *LRRK2* mutations show clinically typical parkinsonism with neuropathology such as substantia nigral degeneration and Lewy bodies (Zimprich et al. 2004). However, in situ hybridization and Northern blotting results revealed surprisingly low signals for *LRRK2* mRNA in brain, especially very low levels in critical PD sites including the SN (Zimprich et al. 2004; Galter et al. 2006).

LRRK2 is highly expressed in human peripheral blood mononuclear cells (PBMCs), especially in CD14^+^ monocytes (Hakimi et al. 2011). Multiple single-nucleotide polymorphisms (SNPs) in the *LRRK2* locus also have been reported to be associated with inflammatory diseases including Crohn’s disease (CD) (Van Limbergen et al. 2009). *LRRK2* is potently induced by type II interferon (IFN-γ) in human immune cells (Gardet et al. 2010; Thevenet et al. 2011) and suppress the activity of the transcription factor Nuclear Factor of Activated T cells (NFAT), which transcriptionally activates the expression of inflammatory genes (Liu et al. 2011; Vora and McGovern 2012). This suggests that LRRK2 provides negative feedback to inflammatory activation upon type II interferon–mediated inflammatory response through degradation of NFAT. IFN-γ is highly upregulated in CD patients, and is involved in the T-helper 1 immune response by CD4^+^ T cells (Fuss et al. 1996; Rafa et al. 2010). Mice lacking LRRK2 exhibit increased sensitivity to the zymozan-induced inflammatory response and experimentally induced inflammatory bowel disease (Liu et al. 2011). Finally, the 2397-M allele of M2397T polymorphism in *LRRK2*, which is linked to sporadic CD cases by GWAS, results in reduced LRRK2 protein expression. All these observation suggested that attenuation of negative feedback system to type II interferon–mediated inflammatory response is potentially involved in the pathogenesis of CD (Liu et al. 2011). The putative role of LRRK2 in immune function raises the possibility that LRRK2 contributes to PD by altering innate immune response to brain pathology. Transcriptional profiles of PBMCs from PD patients carrying the G2019S *LRRK2* mutation show dysregulation in monocytic immune response pathway (Mutez et al. 2011).

In this study, we characterized how LRRK2 M2397T polymorphism affects CD14^+^ mononuclear cell activity in CD. We also demonstrated how LRRK2 kinase activity is responsible for the LRRK2-mediated inflammatory response in CD and PD with *LRRK2* mutations. Our study shows the possibility that LRRK2 modify the disease progression through innate immune response.

## Materials and Methods

### Patient Recruitment and Ethics

**Fig. 1:**
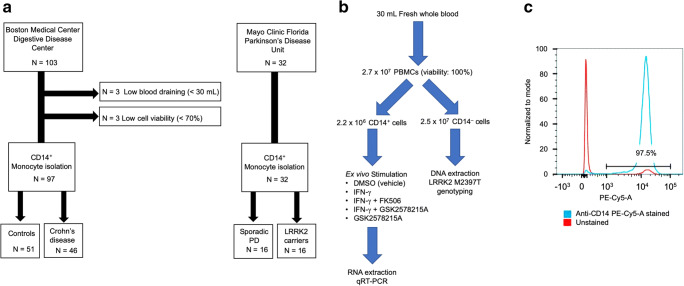
Flow chart of patient recruitment and isolation of CD14^+^ monocytes (A) Flow chart of patient recruitment (B) A flow chart of experimental procedure. DMSO; dimethylsulfoxide, IFNγ: interferon-γ. (C) FACS analysis of isolated CD14^+^ monocytes from peripheral blood. The blue and red lines are for anti-CD14-PE-Cy5-A stained and unstained samples, respectively. The figure shows 97.5% purify of CD14^+^ monocytes after magnetic beads isolation.

After informed consent, 46 Crohn’s disease patients in remission state and 51 non-CD control patients without inflammatory conditions (Boston Medical center) and 16 LRRK2 PD mutant carriers and 16 non-carriers (Mayo Clinic, Florida) were recruited based on the clinical inclusion and exclusion criteria (Tables 1 and 2). During the sample preparation process, 6 cases were excluded due to low blood volume (*n* = 3) and low cell viability (n = 3) (Fig. 1A). The study was approved by the Mayo Clinic and BU Institutional Research Board.

**Table 1:** Clinical inclusion/exclusion criteria for Crohn’s disease

Inclusion criteria	
1. Patients with 5-ASA, corticosteroid, AZA or 6-MP monotherapy	
2. Patients with 5-ASA + corticosteroid, AZA + corticosteroid dual therapy	
3. Age 18–75	
Exclusion criteria	
1. Patients with surgery within the past year	
2. Patients with fissure, ileus or intravenous hyperalimentation treatment	
3. Patients having more than three combinations of drug treatment simultaneously	
4. Patients on anti-TNF-α or MTX treatment	
5. Patients with active inflammation with serum CRP level of higher than 2	
5-ASA: 5-aminosalicylic acid; AZA: azathioprine; 6-MP: 6-mercaptopurine;	

*TNF-α* tumor necrosis factor-α, *MTX* methotrexate

**Table 2:** Clinical exclusion criteria for Parkinson’s disease

Exclusion criteria	
1. Patients with operation history within the past one year	
2. Patients with active inflammation with serum CRP level of higher

### Human Blood Collection and Isolation of CD14^+^ Mononuclear Cells

**Fig. 2:**
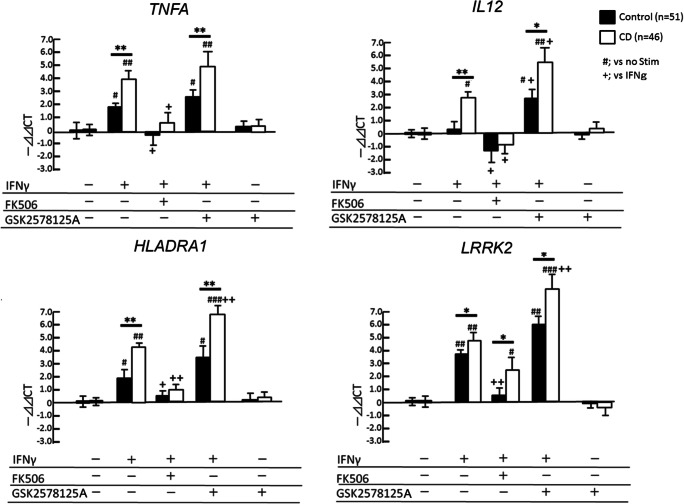
Gene expression analyses of IFN-γ stimulated CD14^+^ monocytes in CD cases. CD14^+^ monocytes from healthy control (Control, *n* = 51) and CD cases (*n* = 46) are subjected to ex vivo stimulation with 100 IU/mL IFN-γ in the presence or absence of FK506 and GSK2578215A (LRRK2-specific kinase inhibitor) for 4 h. The gene expression of *TNFA*, *IL12*, *HLADRA1* and *LRRK2* are examined. * or ** denotes *p* < 0.05 and 0.01 vs. controls with the same ex vivo treatment. ^#^, ^##^ or ^###^ denotes p < 0.05, 0.01 or 0.001 vs. unstimulated group of the same subjects. ^+^ or ^++^ denotes p < 0.05 or 0.01 vs. IFN-γ-stimulated group of the same subjects as determined by one-way ANOVA with Tukey post hoc*.*

Human specimens were handled according to IRB approved institutional guidelines from Boston University School of Medicine and Mayo Clinic Florida. Fresh venous blood (30 mL) were collected using EDTA as an anticoagulant (BD Vacutainer, Franklin Lakes, NJ). The Mayo samples were delivered to the Boston University School of Medicine within 24 h of collection. PBMCs were isolated from the EDTA-treated whole blood by density gradient centrifugation using Ficoll-Paque and Leucosep tubes according to the manufacturer’s instructions (Miltenyi Biotech Inc., Auburn, CA). CD14^+^ mononuclear cells were affinity purified by microbeads by affinity purification by human CD14 microbeads (MACS system, Miltenyi Biotec, Fig. 2A-B).

### Ex Vivo Stimulation

Isolated CD14^+^ mononuclear cells from whole blood samples obtained from Crohn’s and control patients (2.2 × 10^6^ cells/sample) were stimulated by 100 IU/mL IFN-γ (R&D systems) ± 10 ng/mL NFAT inhibitor (FK506, Sigma-Aldrich) or LRRK2 inhibitors (1 μM GSK2578215A, GlaxoSmithKline Pharmaceuticals R&D). The reaction was stopped by addition of Qiazol (Qiagen) at a 1:1 vol ratio and vortexing for 10 s to denature RNase and protect total RNA from degradation, followed by total RNA isolation (Qiagen). Flowchart of CD14^+^ monocyte isolation, stimulation and RNA isolation.

### Quantitative Real-Time Reverse Transcription PCR

Gene expression levels were quantified by real-time reverse transcription PCR (RT-PCR, Mastercycler ep realplex 4s, Eppendorf) using following primers. *TNFA* F: 5-CCCAGGGACCTCTCTCTAATC-3, R: 5-ATGGGCTACAGGCTTGTCACT-3 (Stordeur et al. 2002), *IL12* (p35) F: 5-CCTCAGTTTGGCCAGAAACC-3, 5-GGTCTTTCTGGAGGCCAGGC-3 (Riganò et al. 1999),

*HLADRA1* F: 5-GGACAAAGCCAACCTGGAAA-3, R: 5-AGGACGTTGGGCTCTCTCAG-3 (Corso et al. 2011), *LRRK2*: 5-CTTGGCTTGGTCCTTTATTTCC-3, 5-CCTGAGGCTGTTCCTTCTTCC-3 (Milosevic et al. 2009), *GAPDH* 5-TCATCTCTGCCCCCTCTGCT-3, 5-CGACGCCTGCTTCACCACCT-3 (Gardet et al. 2010).

### *LRRK2* Genotyping

A fraction of CD14 negative PBMCs were subjected to isolation of genomic DNA and PCR direct sequencing of M2397T allele. This is located in exon 9 (rs37618963) and corresponds to a point mutation of coding sequence 7190 T > C (Fig. 2C). PCR primers (PARK8_49F: 5’-CATAATGGTGGTGGTGTCATG-3′,

PARK8_49R: 5’-CTGTGTGACCCTCCAAGACC-3′) produced a 451 bp amplicon for the DNA sequencing of the target site (21).

## Results

### Enhanced IFN-γ Response in CD Group

Since LRRK2 is highly expressed in myeloid cells, we used CD14^+^ monocytes isolated from PBMC using magnetic bead cell isolation. The yield was approximately 2 × 10^6^ CD14^+^ cells from 2.7 × 10^7^ PBMC in 30-mL fresh whole blood with more than 98% purity (Fig. 1B-C). Ex vivo stimulation of CD14^+^ monocytes with IFN-γ induced gene expression of inflammatory cytokines (*TNFA* and *IL12*), major histocompatibility complex molecules (*HLADRA1*), and *LRRK2*, which was signifcantly suppressed by FK506 (Fig. 2), a specific inhibitor of calcineurin and known inhibitor of NFAT pathway via enhancing its phosphorylation-dependent degradation (Liu et al. 2011). We also observed enhanced induction of the tested inflammatory genes and *LRRK2* itself by a potent LRRK2 specific kinase inhibitor (GSK2578215A, Fig. 2) (Reith et al. 2012). Since LRRK2 is known to suppress NFAT signaling via its phosphorylation and proteasomal degradation, inhibition of LRRK2 kinase activity may enhance NFAT-induced gene expression, including *LRRK2* itself. This finding also suggests that IFN-γ induction of LRRK2 is a negative-feedback system to control inflammatory signaling.

### *LRRK2* M2397 Allele Enhances IFN-γ Response in CD Group

**Fig. 3:**
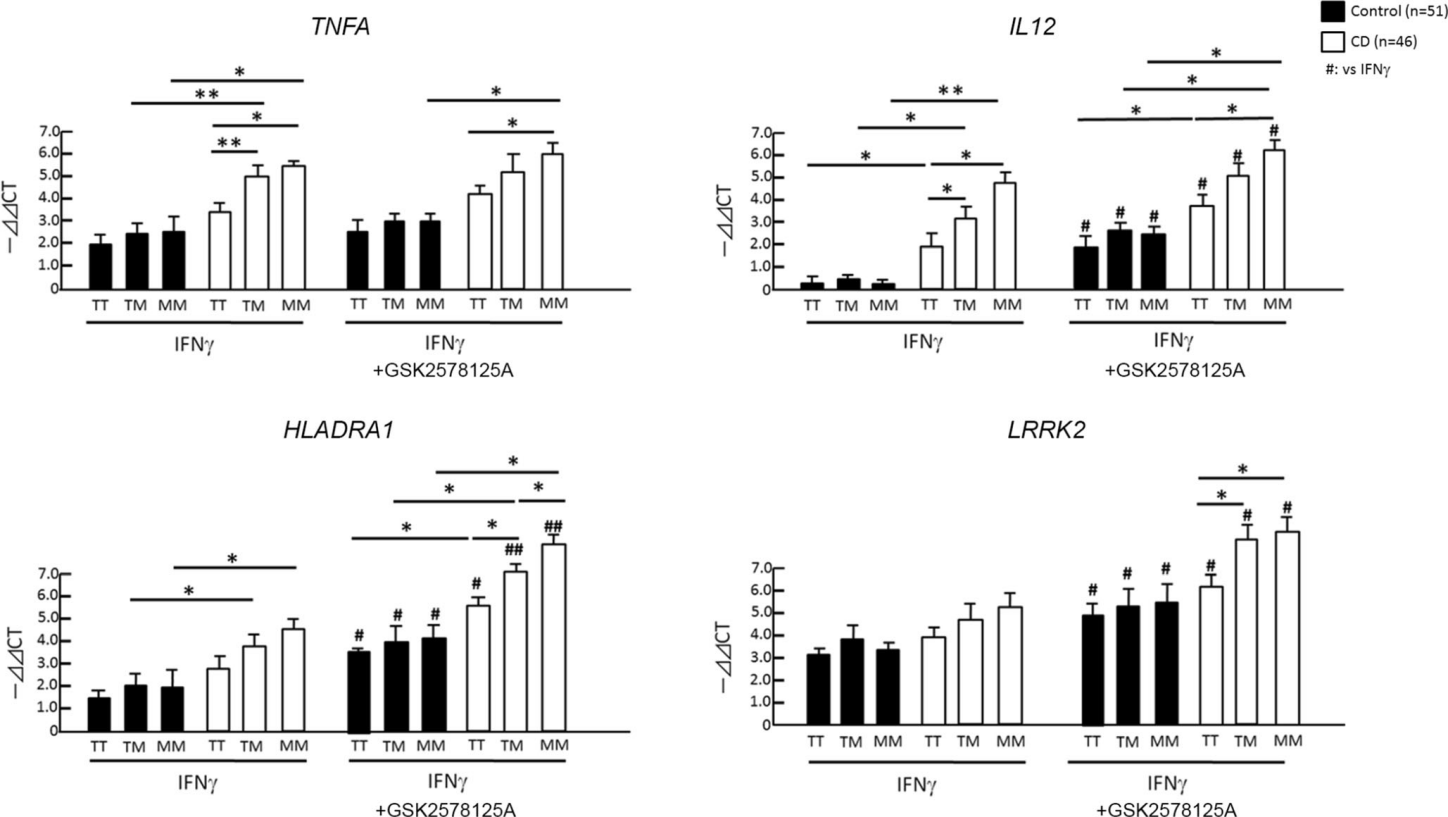
LRRK2 M2397T allele-specific analysis of IFN-γ stimulated CD14^+^ monocytes in CD cases. LRRK2 M2397T allele-specific analysis of CD14^+^ monocyte gene expression of *TNFA*, *IL12*, *HLADRA1* and *LRRK2*. * or ** denotes p < 0.05 and 0.01 vs. healthy control subjects with the same LRRK2 2397 allele and ex vivo treatment and ^#^ or ^##^ denotes p < 0.05 or 0.01 vs. unstimulated group of the same subjects as determined by one-way ANOVA with Tukey post hoc*.*

We have genotyped CD risk SNP (rs37618963) on *LRRK2* in CD and control cases (Table 3). As expected, LRRK2 2397-M allele is more prevalent than 2397-T allele in CD cases as compared to control cases, with odds ratio of 1.44. We compared the IFN-γ-induced gene expression of inflammatory molecules based on the *LRRK2* locus rs37618963 polymorphism. There was no difference in IFN-γ response in control groups among rs37618963 alleles (closed columns, Fig. 3). Strikingly, 2397-T/M and 2397-M/M allele cases show enhanced IFN-γ-induced expression of *TNFA*, *IL12* and *HLADRA1* as compared to 2397-T/T allele in CD cases (open columns) but not in control cases (closed columns, Fig. 3), although *LRRK2* expression was unaltered. GSK2578215A treatment enhanced gene expression of *IL12*, *HLADRA1* and *LRRK2* as compared to IFN-γ alone in both control and CD groups. Although 2397-M allele is shown to reduce LRRK2 protein levels, enhancement of *LRRK2* expression in 2397-T/M and 2397-M/M allele CD cases by GSK2578215A treatment compared to 2397-T/T allele in CD cases (open columns) suggests enhanced LRRK2 kinase activity in 2397-T/M and 2397-M/M allele CD cases, which may result in augmentation of *LRRK2* expression upon its kinase inhibition. These data show that 2397-M allele, a risk allele of CD, is a loss-of-function mutation and enhances IFN-γ-induced inflammatory response in CD group, and GSK2578215A treatment further augment the IFN-γ response both in control and CD groups.

**Table 3:** Demographics of CD and control cases

	Crohn’s (n = 46)	Control (n = 51)
Age	47.26 ± 15.24	42.30 ± 15.20
Gender (M/F)	28/21	30/20
LRRK2 M2397T		
MM	8	4
MT	21	23
TT	17	24
%M allele	40.22*	30.39

*Odds ratio 1.44

### IFN-γ Response in CD14^+^ Monocytes from Sporadic PD and PD-Linked* LRRK2*-Mutated Cases

**Fig. 4:**
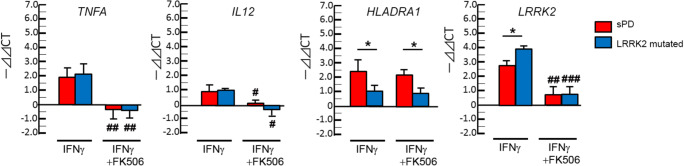
Gene expression analyses of CD14^+^ monocytes in PD cases. CD14^+^ monocytes isolated from sporadic PD (sPD, *n* = 16) and PD-linked LRRK2 mutated cases (n = 16) were analyzed for gene expression of *TNFA, IL12, HLADRA1* and *LRRK2*. * denotes p < 0.05 vs. sPD with the same ex vivo treatment and ^#^, ^##^ or ^###^ denotes p < 0.05, 0.01 or 0.001 vs. unstimulated group of the same subjects as determined by one-way ANOVA with Tukey post hoc*.*

We next performed a similar study using CD14^+^ monocytes isolated from sporadic PD (sPD) and PD-linked LRRK2-mutated (G2019S and R1441C) cases (Zimprich et al. 2004; Nandhagopal et al. 2008). Patients characteristics including LRRK2 M2397T polymorphism were shown in Table 4. Due to the limited sample volume per donor, we focused on the IFN-γ response in the presence or absence of FK506 in this cohort. IFN-γ-induced expression of *TNFA* and *IL12* expression was unchanged between sPD (red column) and *LRRK2*-mutated cases (blue column), although both show potent suppression by FK506 (Fig. 4). Surprisingly, IFN-γ-induced expression of *HLADRA1* levels were lower in *LRRK2*-mutated cases as compared to sPD cases, and is insensitive to FK506 treatment. IFN-γ-induced expression of *LRRK2* was significantly enhanced in *LRRK2*-mutated cases as compared to sPD cases and sensitive to FK506. In contrary to CD-associated *LRRK2* mutations, PD-linked *LRRK2* mutations show little effect to IFN-γ-induced gene expression. This was unexpected, since PD-linked G2019S LRRK2 mutation is known to enhance is kinase activity (Kelly et al. 2018), which would suppress inflammatory response and *LRRK2* expression. The data suggests development of compensatory mechanism for IFN-γ-induced gene expression in PD-linked *LRRK2* mutated cases.

**Table 4:** Demophrahics of sPD cases and LRRK2 mutant carriers

	sPD (n = 16)	LRRK2 mutant	
		PD (*n* = 6)	Carrier (*n* = 10)
Age	67.06 ± 12.26	71.67 ± 9.95	50.50 ± 10.05**
Gender (M/F)	8/8	4/2	5/5
Lrrk2 M2397T			
MM	2	0	1
MT	7	3	6
TT	7	3	3
%M allele	34.37	25	40

**p < 0.01 vs. LRRK2 mutant PD

## Discussion

In this study, we have tested the effect of the *LRRK2* mutations associated with CD and PD using freshly isolated human CD14^+^ blood monocytes for ex vivo stimulation with IFN-γ under the influence of NFAT-specific inhibitor (FK506) or LRRK2-specific kinase inhibitor (GSK2578215A).

In the CD Study, our findings are 1) IFN-γ response induces gene expression of *TNFA, IL12, HLADRA1* and *LRRK2* in CD14^+^ monocytes, 2) GSK2578215A enhances IFN-γ responses, while NFAT suppresses, 3) LRRK2 2397-M group show enhanced IFN-γ responses in CD group but not in the control groups, and 4) GSK2578215A further enhances IFN-γ responses regardless of M2397T allele. These data substantiate our hypothesis that LRRK2 is induced by IFN-γ and LRRK2 inhibition would enhance inflammatory gene expression. This function is impaired by CD-linked LRRK2 mutation in CD cases, presumably due to the nonsense RNA decay of *LRRK2*. Our recent study of the occurrence of CD with PD cohort identified only 2 cases of co-occurrence out 876 PD patients (0.2%), which is within the known prevalence of CD (26.0–198.5 cases per 100,000 persons) (Fujioka et al. 2017). A larger sample size is necessary to evaluate the effect of LRRK2 M2397M allele on the co-occurrence of CD and PD as a future study.

In the PD study, our findings are 1) IFN-γ-induced *TNFA* and *IL12* expression was similar and sensitive to FK506 in sPD and *LRRK2 *mutated groups, 2) IFN-γ-induced *HLADRA1* expression was reduced in *LRRK2* mutated group and was insensitive to FK506, and 3) IFN-γ-induced *LRRK2* expression was unexpectedly increased in *LRRK2*-mutated group and was sensitive to FK506. This suggests that PD-linked *LRRK2* mutation may not have consistent effect for modulating IFN-γ-induced immune response as expected, but may affect antigen presentation in myeloid cells. Further study will be necessary to test the effect of ex vivo or systemic administration of LRRK2 inhibitors on enhancing *HLADRA1* expression and antigen presentation machinery ex vivo or in vivo for evaluating its clinical application. For CD cases with 2397-M/M allele, specific LRRK2 activator may be beneficial for compensating the reduced activity of LRRK2 in myeloid cells in case chronic treatment with FK506 is inapplicable. In such a case, the drug would need a careful evaluation for the off-target effect of LRRK2 in other organs, such as kidney which have a high expression of LRRK2.
